# Collaboration among psychological researchers, the government, and non-profit organizations for “Konkatsu” (marriage hunting) in Japan

**DOI:** 10.3389/fpsyg.2022.982102

**Published:** 2022-09-20

**Authors:** Takashi Nishimura, Toshihiko Souma, Mie Kito, Junichi Taniguchi, Yuji Kanemasa, Junko Yamada, Yuki Miyagawa

**Affiliations:** ^1^Department of Psychology, Hiroshima International University, Higashi-Hiroshima, Japan; ^2^Graduate School of Humanities and Social Sciences, Hiroshima University, Hiroshima, Japan; ^3^Department of Sociology, Meiji Gakuin University, Tokyo, Japan; ^4^Department of Psycology, Tezukayama University, Nara, Japan; ^5^Faculty of Psychology, Otemon Gakuin University, Ibaraki, Japan; ^6^Department of Psychology, Rissho University, Tokyo, Japan; ^7^Brain Science Institute, Tamagawa University, Tokyo, Japan

**Keywords:** konkatsu (marriage hunting), collaboration with government and non-profit organizations, social implementation, collaborative research, physical attractiveness, the decline in the number of marriages in Japan

## Abstract

In contemporary Japanese society, it is difficult to find a marriage partner, and therefore, “Konkatsu,” the search for a marriage partner, has become a socially accepted activity in Japan. In response to this social challenge, in addition to private companies, governments and non-profit organizations are supporting individuals in their search for a marriage partner. This paper reviews statistical information related to marriage hunting published in Japan. In addition, some of the authors’ collaborative activities and academic publications based on these activities are reviewed. Subsequently, the paper discusses and highlights the importance of helping individuals have confidence in their physical attractiveness.

## Introduction

Modern-day Japan is facing the problems associated with a declining birthrate and aging population. As a part of the social measures undertaken to address such problems, the Japanese government provides support for the implementation and operation of “marriage hunting,” or the activity of finding a marriage partner. In difficulties in finding a partner due to social changes, such as the decline of arranged (*omiai*) marriages, not only private companies, but also governments and non-profit organizations, are supporting individuals in their search for a partner by providing young people opportunities to meet a potential partner, to go on social dating, and to get married eventually. Due to a lack of empirical studies on marriage hunting events, many questions remain unanswered: Why do people participate or not participate in the events of marriage hunting? What are the psychological processes behind the decision-making leading to matching in such events? In this paper, we (1) describe the marriage hunting support currently provided by the Japanese government based on government statistics and the literature review and (2) discuss our attempts for collaborating with the government and non-profit organizations in the context of marriage hunting. We then (3) describe the results of our exploratory study among “marriage hunters” who have never participated in the related events (Nishimura et al., 2019), and (4) argue, based on our findings, that one of the potential future directions in supporting the activity of marriage hunting is to increase participants’ confidence in their physical attractiveness. In addition, we will discuss the need to address structural and subjective barriers to participation in marriage-hunting events in Japan.

## Marriage hunting support provided by the Japanese government

The decline in the number of marriages and the resultant low birth rate are expected to have various effects on the future of Japanese society. Therefore, this is an urgent issue that must be addressed. There has been a prominent decrease in the number of marriages post-2020, given the various behavioral restrictions implemented to prevent the spread of COVID-19. According to the Vital Statistics published by the Ministry of Health, Labor, and Welfare (2022), the number of weddings conducted between March 2019 and February 2020 was 639,690, which dropped to 527,446 from March 2021 to February 2022, a decrease of 17.5% from the previous year. According to the National Institute of Population and Social Security Research (2022), the lifetime unmarried rate (the percentage of unmarried people who are aged 50) was 28.25% for men and 17.81% for women in 2020, and this rate has not decreased in recent years.

Yamada and Shirakawa (2008) proposed “konkatsu” (marriage hunting) as a concept to describe social movements related to marriage in Japan. This process involves various steps such as registering with a marriage agency, attending events and parties with multiple people in attendance, meeting somebody from the opposite sex after being matched *via* various registration data, attending seminars to raise one’s awareness of marriage, and engaging in activities to enhance one’s own attractiveness (for example, undertaking self-care efforts by visiting a beauty salon).

Yamada and Shirakawa (2008) stated that single individuals need to undertake additional steps to get married. These include investing in themselves to increase their attractiveness, improving communication skills, creating opportunities to meet members of the opposite sex, and adjusting their ideal conditions for marriage (e.g., ideal economic status). The advocacy of this concept was combined with the measures undertaken by local governments to combat the problem of a declining birthrate, and accordingly, many local governments have increasingly implemented government-supported marriage-hunting programs. The administrative efforts undertaken in this regard were summarized by Otaki (2010) and Bin (2015).

In addition, according to the Cabinet Office (2022), the most notable effect estimated by local governments (prefectures) implementing interventions to prevent fertility decline was “an increase in the total fertility rate (93.6%)” followed by “an increase in the number of marriages (87.2%).” In Japan, support for marriage is being provided as part of measures to combat the declining birthrate. According to the Cabinet Office (2022), local governments across Japan are implementing various marriage-hunting initiatives. Among 47 prefectures, 74.5% were involved in the “establishment and operation of marriage support centers,” 70.2% held in “marriage-hunting events,” and 66.0% were involved in “encouraging registration at marriage support centers.” In addition, 63.8% of the prefectures conducted “skill improvement seminars for those who wish to get married,” 61.7% were involved in the “establishment and operation of a matching system,” and 59.6% carried out the “training and organization of marriage support volunteers.” Such efforts indicate that the objective is not only to set up a system for marriage hunting but also to undertake initiatives that provide direct or indirect psychological support to the participants.

How are local governments implementing such efforts in cooperation with private organizations? According to the Cabinet Office (2022), 75.8% of the prefectures (25 out of 33) collaborated with private organizations for marriage-hunting events. Among the private organizations, 66.7% were companies, 27.3% were incorporated associations and foundations, 24.2% were local organizations such as agricultural cooperatives, 15.2% were non-profit organizations, 6.1% were social welfare councils, fishing cooperatives, and commerce and industry associations, and 6.1% were labor-management organizations and labor bureaus. However, universities and other research institutions were not set as categories for such partner organizations. The survey’s options and actual results suggest the following points. First, private company-led event implementation is more likely to be chosen as a collaboration partner for the government. Second, it is not always common for government and research institutions to collaborate regarding “marriage activities.”

Thus, it is not common for a particular group of academic researchers (especially social psychologists who study interpersonal attraction) and the government to collaborate in activities that support marriage hunting in Japan. However, we have conducted several projects on marriage hunting in collaboration with administrative organizations in Hiroshima Prefecture. We saw the cooperation as bringing benefits to both sides. On the one hand, the government is able to verify the effectiveness of marriage hunting support programs based on scientific evidence. On the other hand, researchers can conduct research on interpersonal attraction based on data with high ecological validity. In the following section, we describe the details of our collaborative projects.

## Our attempts for collaboration with the government and non-profit organizations

In the context of psychological research, interpersonal attraction and partner selection in marriage hunting can be examined using speed dating methods. Speed dating is a method through which real-life dating situations can be replicated (Finkel et al., 2007).

The most crucial issue in implementing this method with a team consisting only of academic researchers without government collaboration is the difficulty of gathering an equal number of male and female participants interested in marriage hunting. In addition, it is common for marriage-hunting parties conducted by governments and private companies to incorporate speed dating methods in their proceedings. For this reason, our research team collaborated with the “*Koi no Wa*” (Ring of Love) Project, which is now run by the Hiroshima Foundation for Prospect of Children, since 2019. We organized an event called the “Marriage-Hunting Event Held by Social Psychologists.” The event was publicized on a dedicated website of “Koi no Wa” and participants were recruited from the “Koi no Wa” registered monitors. In general, the participation fee usually charged at similar face-to-face events is ￥4,000 for men and ￥1,000 for women; this covers the cost of the venue and the food and drinks provided at the event. However, as our research group was able to conduct the event using university facilities, we set the participation fee at ￥1,000 (about USD9 at the time of the event) for both men and women to cover the actual cost of refreshments provided at the event, which is lower than the participation fee charged at other similar events. It would have been possible to hold the event with no participation fee, but we considered the risk of cancellations on the day of the event and decided to collect the participation fee. Many people expressed their interest in participating in the event, given the low participation fee and the fact that social psychologists would provide suggestions to participants from a psychological perspective. The number of applicants exceeded the maximum number of participants allowed for all three events held between November and December 2019. Each session included a quota of 20 males and 20 females. However, 33 men and 47 women were included in the first session, 38 men and 45 women in the second, and 31 men and 34 women in the third. The government (“*Koi no Wa*” secretariat) selected the participants after confirming that they met the application requirements (e.g., previous participation count) and notified the applicants of their acceptance or rejection. Because the program was conducted with the cooperation of the government, the participants were personally contacted by the secretariat when necessary.

In addition, Hiroshima Prefecture reports the extent to which dating and marriage-hunting support measures have led to successful marriages (Hiroshima Prefecture, 2020). The Hiroshima Dating Support Center saw 200 couples marry in FY 2019. The total number of marriages in Hiroshima Prefecture was 13,185 in 2019. Therefore, the percentage of marriages in Hiroshima Prefecture that occurred because of government initiatives is approximately 1.5%. The direct effect of this initiative may seem low, as various factors have a combined effect on the process from actual encounters to marriage. However, a private company called IBJ Inc., which operates one of Japan’s largest marriage agency networks, reported that the number of marriages through that company reached a record high of 10,402 in 2021, equivalent to 2% of the total number of marriages in Japan (IBJ, Inc., 2022). If we consider this number as an indicator, it can be said that marriage-hunting support by the Hiroshima Dating Support Center has significantly contributed to the number of successful marriages. Hence, the government and researchers’ efforts to support marriage hunting are important in creating marriage opportunities. As mentioned, the number of marriages in Japan has been rapidly declining. The situations that inhibited interaction with others implemented during the COVID-19 pandemic decreased opportunities for social interaction. This is expected to accelerate a decline in the number of marriages in the future. Addressing this situation is a pressing issue faced by Japanese society.

Furthermore, it has been pointed out that, concerning marriage support, romantic relationship support is needed to enhance romantic feelings as a preliminary step to marriage support. Based on a text-mining analysis of general-interest magazines from the 1970s to the 2000s, Tanimoto and Watanabe (2016) found that the romantic love ideology, which suggests that romantic love should be legitimized by marriage (mediated by a matchmaker), weakened in the 1980s and shifted to a romantic marriage ideology in the 1990s, according to which marriage should be legitimized by love. Takahashi (2021) argued that, particularly for men, overcoming the lack of ideal economic status that women seek in men through romantic love may be a possible strategy for achieving success in the marriage-hunting market. Economic disparities between men and women in the marriage market are structural concerns that would be difficult to resolve. Therefore, it would be crucial to encourage the development of romantic feelings by providing support to facilitate this process and by increasing confidence in their physical attractiveness.

Kobayashi and Nochi (2016) also presented the data obtained from the registrants of marriage support in Ehime Prefecture for a marriage support program conducted in cooperation with the government. The results of the analysis indicated the following four points: (1) For men, the chances of marriage increased with higher socioeconomic status in terms of education, full-time employment, and income. (2) For women, their socioeconomic statuses had no such effect. (3) For both men and women, the chances of marriage increased with age and marital experience. (4) The chance of marriage increased for tall men. The only appearance-related variable included in the analyses is height, and it is unclear whether other appearance factors affected individuals’ chances of marriage. Through these data analyses, Kobayashi and Nochi (2016) pointed out the importance of providing relationship support, which emerged as a preliminary step to providing marriage support. In other words, it becomes difficult to get married without romantic relationship support. Thus, the necessity of establishing a mechanism to allow people to naturally develop romantic feelings toward their partners in marriage support situations is evident.

## Negative impact of low confidence in physical attractiveness on marriage hunting

What factors might inhibit participation in marriage-hunting situations? It is difficult to determine a solution to this question for people who have no desire to get married in the first place. However, it is possible to investigate the factors that may inhibit people from participating in marriage hunting by asking those who have registered for the government’s marriage-hunting support program but have never actually participated in marriage-hunting events. In this regard, Nishimura et al. (2019) reported the results of their analysis of data obtained from people who had registered for the marriage hunting support program run by Hiroshima Prefecture but had never actually participated in the event. In some dating services managed mainly by local governments, people register as monitors to obtain information related to marriage hunting and its events; the registered monitors may apply to participate in the events. However, not all registered individuals actively participate in these events. As of June 2019, approximately 14,000 people had registered as “*Koi no Wa*” monitors in Hiroshima prefecture, but among them, many were “dormant members” who had never participated in any events. Increasing the commitment to marriage by hunting for people who are interested in marriage and are registered in events and connecting them to event participation could increase the marriage rate in the long run. Therefore, we conducted an exploratory study considering the determinants of willingness to participate based on the attitudes and mindset of these “dormant members.”

The outline of Nishimura et al.’s (2019) method is as follows: An online survey was conducted in March 2019 with those registered with dating services managed primarily by local authorities. Participation in this survey was completely voluntary. This survey sought to ascertain the current status of the monitors’ activities and their attitudes toward marriage. It was unrelated to a specific event. Therefore, respondents came from a variety of backgrounds regarding marriage hunting. A total of 802 participants completed the survey. Among them, 213 respondents who were unmarried and had “never attended any of the events listed on the Support Center website after registering as a member” were included in the analysis.

When asked, “would you like to participate in future events organized by the service?” using a 4-point scale, 85.9% of the total number of respondents answered “strongly agree (4)” and “agree (3).” This shows that dormant members would be highly likely to participate in marriage-hunting events if their conditions were met. Table 1 shows the frequency and percentage of the reasons respondents selected for not attending previous events, as well as the ranking of the options selected. Both external reasons, such as “I am too busy with work” and “I did not get selected even after applying for it,” and internal reasons, such as “lack of self-confidence” and “I feel uncertain,” were highly selected.

**Table 1:** Frequency, percentage, and ranking of reasons for non-participation by those who are registered but have not participated in the event.

	Ranking	Frequency	Percentage
I am too busy with work	1	61	28.6
I lack self-confidence (e.g., appearance)	2	59	27.7
I do not have an event I want to attend	3	49	23.0
I did not get selected even after applying for it	4	45	21.1
I feel uncertain	5	44	20.7
I’ll be shocked if no one picks me	6	40	18.8
I’m nervous	7	39	18.3
I do not have the courage to go to events	7	39	18.3
I’m not good at talking to the opposite sex	9	28	13.1
I do not know how to participate	10	27	12.7
The participation fee was too high	10	27	12.7
I do not know what kind of people will participate in the event until I attend it	12	22	10.3
I hope to meet someone naturally rather than at an event	13	21	9.9
I’m afraid of matching with a member of the opposite sex that I am meeting for the first time	14	17	8.0
I’m afraid that there will be no one I like	15	13	6.1
No reason	16	8	3.8
I do not want to take time out of my personal life	17	5	2.3

Source: Nishimura et al. (2019).

This suggests that, in order to encourage those who have registered for an event but are yet to attend it to take the “first step,” it is necessary to make it easier for them to attend an event as well as to increase their confidence in their internal characteristics, such as in the context of their physical attractiveness and personality. Considering these results, those who hesitate to participate in marriage-hunting events need to be encouraged to increase their confidence in the perception of their attractiveness. Strategies to eliminate barriers to participation in marriage-hunting would include pre-conducting communication skills courses and make-up courses.

## Discussion

Research conducted in collaboration with the government, which also provides marriage-hunting support, has advantages for the researcher, participants, and the government (society). There are many effects generated by this collaboration, such as verification of the effectiveness of the intervention measures for marriage-hunting participants who lack confidence in their physical attractiveness, which was examined in this study. In the future, as the number of cases of close cooperation between researchers and the government increases, social issues, such as declining marriages later in life, are expected to be mitigated, and the number of marriages among older adults is expected to increase. Economic disparities between men and women in the marriage market are structural concerns that would be difficult to resolve. However, offering suggestions to the government to address the structural problems underlying marriage hunting is essential. Therefore, researchers and the government could benefit from collaborating to organize marriage hunting events.

Furthermore, to lower the hurdle to participation and encourage continued participation, it is necessary to provide pre-intervention support to participants to increase their confidence regarding appearance attractiveness and improve their social skills for interpersonal relationship development. Specifically, the intervention included a makeup course and small communication courses. In particular, the small courses offered on communication are where a psychologist’s expertise can be demonstrated. Some possible approaches would be to present online videos on how to apply makeup and engage in non-verbal communication before and after registration or to include a communication course before the actual marriage-hunting event takes place.

In addition, given the development of today’s information and communication technology, it is common to communicate using smartphones and personal computers using cameras and microphones. This can also be used in the speed-dating method of marriage hunting. By introducing the rule of voice-only interaction without the camera at the beginning of speed-dating, for example, participants can interact without being overly conscious of their attractiveness.

This study discussed the need to address structural and subjective barriers to participation in marriage-hunting events in Japan. Contributing to the elimination of the psychological obstacles of involvement in marital activities is essential if we are to address the pressing societal issue of population decline in Japan today.

## Data availability statement

The original contributions presented in the study are included in the article/supplementary material, further inquiries can be directed to the corresponding author.

## Ethics statement

Ethical review and approval was not required for the study on human participants in accordance with the local legislation and institutional requirements. Written informed consent for participation was not required for this study in accordance with the national legislation and the institutional requirements.

## Author contributions

TN, TS, and MK contributed to the initial conception and design of this paper. JT, YK, JY, and YM provided advice on the initial conception of this paper from multiple perspectives. All authors participated in the collaboration with the “*Koi no Wa*” project to conduct speed dating. TN wrote the first draft of the manuscript. TS, MK, JT, YK, JY, and YM contributed to the revision of the manuscript, and read and approved its submitted version. All authors contributed to the article and approved the submitted version.

## Funding

This work was supported by the Research Promotion Council for Chugoku Regional Economies, and JSPS KAKENHI Grant Numbers 21 K02973 and 21 K02344.

## Conflict of interest

The authors declare that the research was conducted in the absence of any commercial or financial relationships that could be construed as a potential conflict of interest.

## Publisher’s note

All claims expressed in this article are solely those of the authors and do not necessarily represent those of their affiliated organizations, or those of the publisher, the editors and the reviewers. Any product that may be evaluated in this article, or claim that may be made by its manufacturer, is not guaranteed or endorsed by the publisher.
